# Multiple Xanthogranulomas in a Teenager

**DOI:** 10.7759/cureus.9516

**Published:** 2020-08-02

**Authors:** Katelyn Urban, Miesha Merati, Lydia Parker, Carolyn Sok, Rick Bains

**Affiliations:** 1 Dermatology, Lake Erie College of Osteopathic Medicine, Greensburg, USA; 2 Dermatology, The Parker Skin and Aesthetic Clinic, Beachwood, USA; 3 Pathology, AmeriPath, Cleveland, USA; 4 Pathology, AmeriPath, Indianapolis, USA

**Keywords:** juvenile xanthogranuloma, multiple xanthogranuloma, non-langerhans cell histiocytosis, nevoxanthoendothelioma

## Abstract

Juvenile xanthogranuloma (JXG) is a non-Langerhans cell histiocytosis that typically presents as a solitary lesion in infancy. Multiple lesions, especially in patients over one year of age, are rarely described in the literature. The authors report a case of a 17-year-old female who presented with multiple asymptomatic nodules and plaques. The diagnosis of xanthogranuloma was confirmed with histopathologic examination of foamy histiocytes and the characteristic Touton giant cells. The expected course of multiple JXG in older patients may differ from those presenting with a solitary lesion earlier in life.

## Introduction

Juvenile xanthogranuloma (JXG), the most common non-Langerhans cell histiocytosis, is characterized by a solitary, well-demarcated, firm, red to yellow-brown papule or nodule. Up to 70% of cases present before one year of age [1]. Lesions are commonly located on the face, trunk, and extremities, although any location including extracutaneous sites may be involved [2,3]. Multiple lesions are rare; a 2003 study of 174 cases reported multiple lesions in 7% of patients [1]. The association with neurofibromatosis type 1 and juvenile myelomonocytic leukemia is higher in patients with multiple lesions compared to those with a solitary lesion. There is a male predominance across all presentations of the disease [3]. Self-resolution does occur, but with lesser frequency in older patients with multiple lesions [2]. Histologic examination reveals mixed infiltrate of mononuclear cells and multinucleated giant cells with the Touton-type giant cells appearing in 95% of cases [2,4]. Immunohistochemistry is positive for CD68 and often factor XIIIa [2]. JXG is differentiated from Langerhans cell histiocytosis with a negative S-100 protein and CD1a [4]. 

## Case presentation

**Figure 1:**
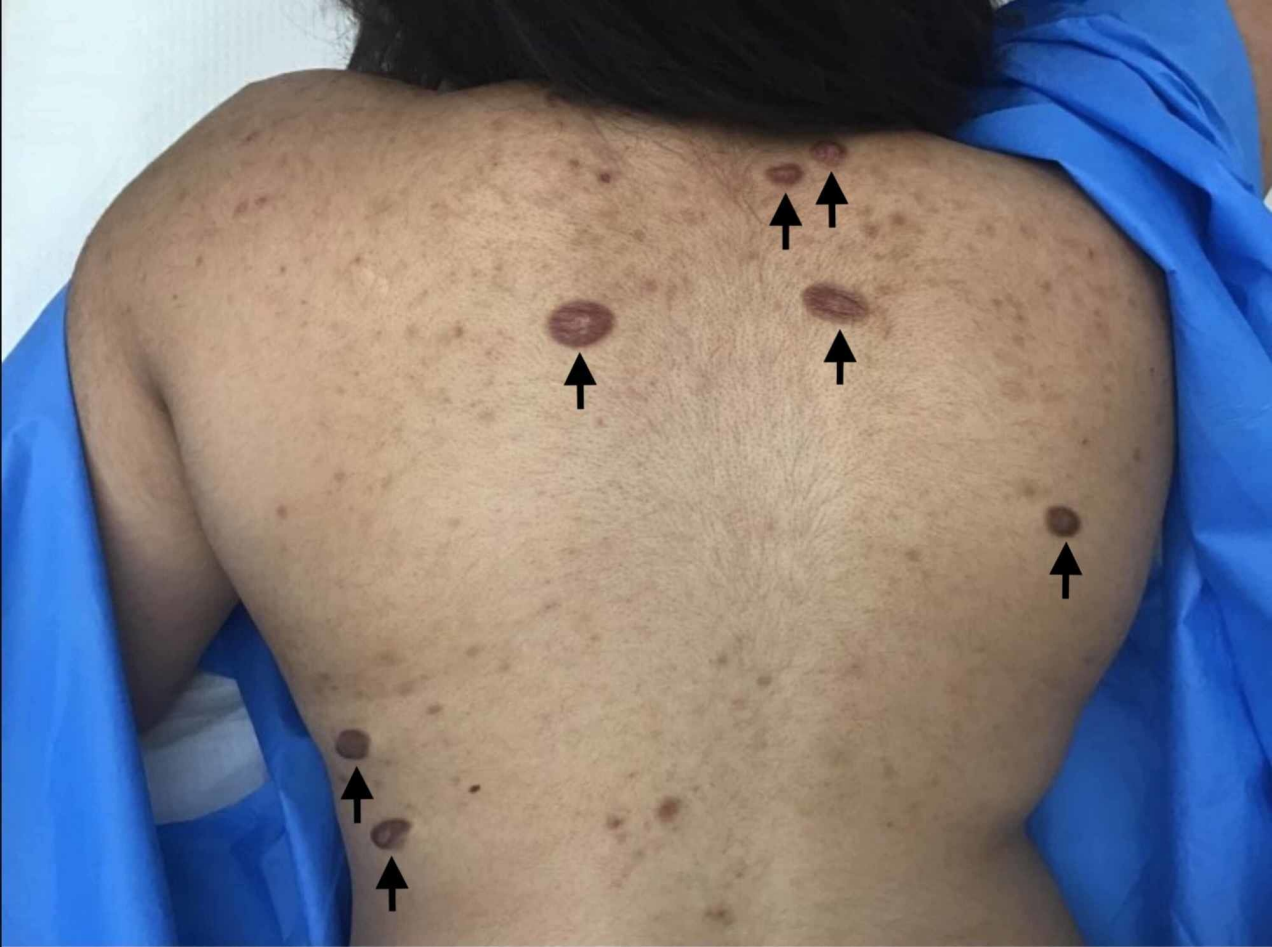
Clinical exam reveals truncal pink to brown firm nodules and atrophic plaques.

A 17-year-old female with no significant past medical history presented with asymptomatic truncal pink to brown firm nodules and atrophic plaques of two-year duration (Figure 1).

**Figure 2:**
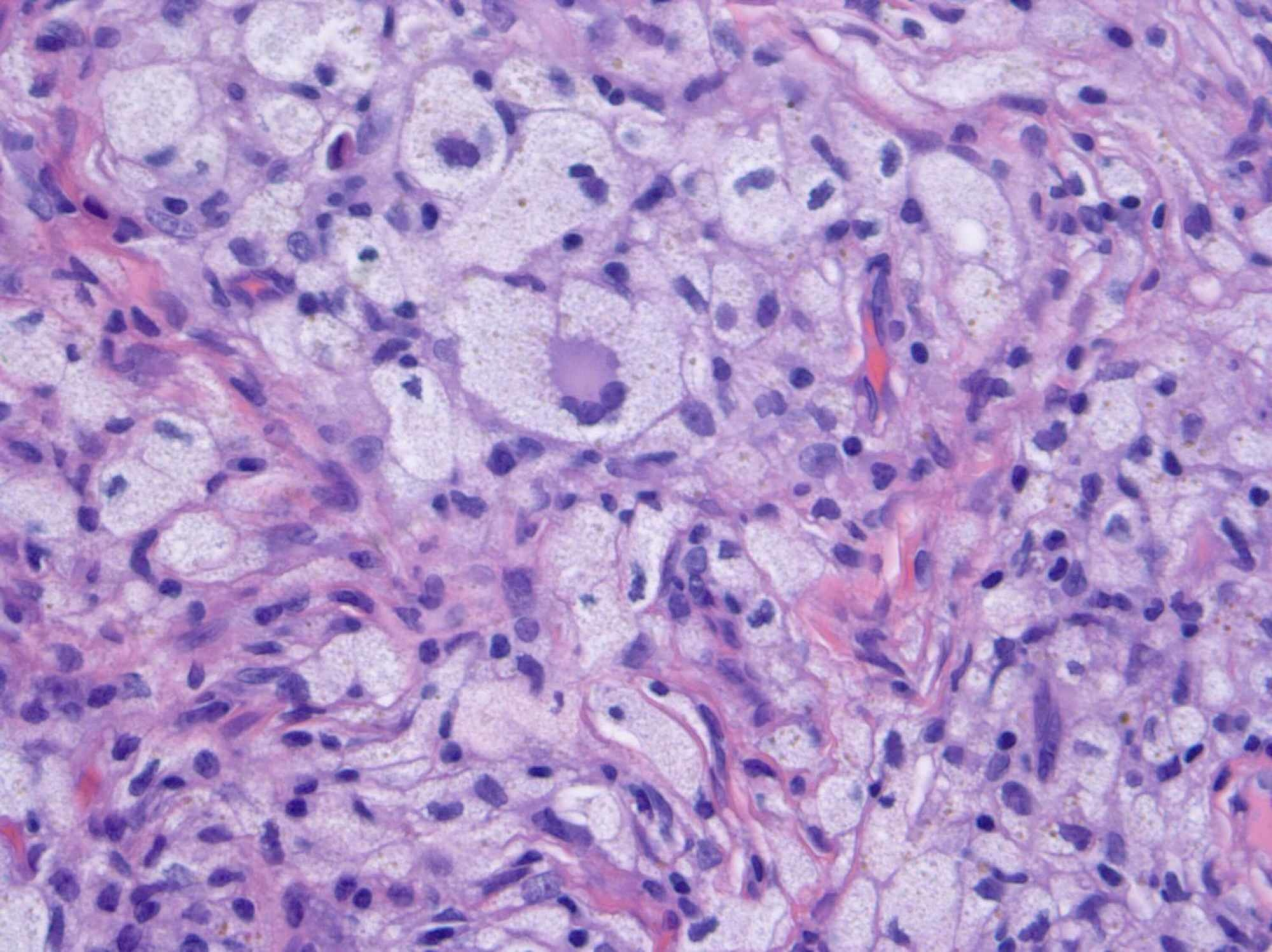
Histopathologic examination of the lesion showing Touton giant cells (hematoxylin-eosin, original magnification ×40).

Prior to presentation, an outside dermatologist treated the lesions as presumed keloids with intralesional kenalog. Two punch biopsies revealed a dense proliferation of foamy histiocytes with multinucleation and Touton giant cells, consistent with xanthogranuloma (Figure 2). Bilobed atypical nuclei, characteristic of Langerhans cell histiocytosis, were not present.

A full body skin exam was only significant for facial and truncal acne, as well as benign nevi, and there were no specific skin findings of neurofibromatosis, as well as no appreciable lymphadenopathy. A complete metabolic panel was normal, and complete blood count revealed anemia, for which the patient was placed on iron supplementation by her primary care physician. The patient had a negative review of systems, and visits with oncology as well as ophthalmology were without concern. On several follow-ups over the span of a year, several lesions resolved with hyperpigmentation; however, many remain. Isotretinoin for her acne, as well as potential incidental improvement of her condition, was offered; however, the patient declined.

## Discussion

Reviewing the literature, we found five cases (three male, two female) of multiple xanthogranuloma with an onset occurring between 13 and 19 years of age. Clinical appearance and histology were consistent with the typical findings of JXG across all cases. Yellow to brown papules and nodules were the most frequently reported description of lesions [1,3-6]. No cases revealed pink to brown plaques as seen in our patient. The brown color may in part be attributed to atrophy and post-inflammatory hyperpigmentation, as a prior dermatologist was injecting kenalog. Focal lentiginous features overlying the histiocytic proliferation were seen on histopathological examination, although a majority of the epidermis appeared attenuated. Additionally, the lesions of JXG may appear darker in patients with Fitzpatrick skin types IV, V, and VI [7].

Systemic JXG was present in a patient undergoing chemotherapy for leukemia at the onset of the lesions [4]. The remaining cases were limited to the skin and were associated with normal hematological studies. Most common locations of the lesions were on the extremities, trunk, and face. Treatment course varied among the cases [1,3-6]. Routine follow-up was elected for two patients, given the benign nature of the disease and tendency for self-resolution [1,5]. Two patients were reported to have spontaneous regression of some lesions [5,6]. A 17-year-old male was treated with isotretinoin, which was discontinued after two months due to surprising lesion progression. Reduction in number and size of the lesions was observed after a year [6]. CO_2_ laser was used in a case with no recurrence of the lesions after five years [3].

JXG is typically benign but requires routine surveillance because of the association with systemic malignancy. Ophthalmology examination is recommended to avoid the significant morbidity associated with an extracutaneous lesion of the eye [1]. Surgical excision, CO_2_ laser, and isotretinoin have been reported with variable response in the treatment of cutaneous lesions, and self-resolution is common in singular lesions, but more rare in multiple [2,3,6].

## Conclusions

A 17-year-old female with multiple xanthogranulomas is an atypical presentation of JXG in terms of age of onset, gender of the patient, and presence of multiple lesions. We report this case to bring attention to the challenging diagnosis with a clinical course that deviates from the expected self-limited nature of solitary lesions. Increased association with systemic involvement and lesion persistence may be considered in the evaluation and management of multiple JXG.
